# Fabrication of Antibacterial Poly(ethylene terephthalate)/Graphene Nanocomposite Fibers by In Situ Polymerization for Fruit Preservation

**DOI:** 10.3390/molecules30153109

**Published:** 2025-07-24

**Authors:** Jiarui Wu, Qinhan Chen, Aobin Han, Min Liu, Wenhuan Zhong, Xiaojue Shao, Yan Jiang, Jing Lin, Zhenyang Luo, Jie Yang, Gefei Li

**Affiliations:** 1 College of Science, Nanjing Forestry University, Long Pan Road No. 159, Nanjing 210037, China; ruijiawu@njfu.edu.cn (J.W.); cqh.vict1238@njfu.edu.cn (Q.C.); hab.oslair@njfu.edu.cn (A.H.); liumin@njfu.edu.cn (M.L.); 15850796961@njfu.edu.cn (W.Z.); luozhenyang@njfu.edu.cn (Z.L.); 2 Highbery New Nano Materials Technology Co., Ltd., Changzhou 213000, China; sxj@highbery.cn (X.S.); jy@highbery.cn (Y.J.); 3 Nanjing Institute of Metrological Supervision and Testing, Ma Qun Avenue No. 10, Nanjing 210049, China; linjing009@126.com

**Keywords:** graphene, nanocomposite fabric, in situ polymerization, food preservation

## Abstract

A novel polyester/graphene nanocomposite fiber was produced using the in situ polymerization protocol with carboxylated graphene and melt spinning technology. The resulting nanocomposite fibers were characterized by X-ray diffraction (XRD), Raman spectroscopy, differential scanning calorimeter (DSC), and scanning electron microscope (SEM). The fibers containing 0.2 wt% graphene fraction showed an excellent dispersity of graphene nanosheets in polymeric matrix. DSC test showed that the efficient polymer-chain grafting depresses the crystallization of PET chains. This graphene-contained PET fabric exhibited attractive antibacterial properties that can be employed in fruit preservation to ensure food safety.

## 1. Introduction

Graphene-polymer nanocomposites have drawn much attention, from laboratory research to industrial production, due to their unique properties [1]. The 2D nanosheet structures give traditional polymer materials many new features such as improved strength, electroconductivity, thermal conductivity, low-temperature toughness, and anti-bacterial properties. However, the extensive applications of graphene-hybrid materials are limited to uniformly dispersed graphene within a polymer matrix [2]. The traditional melt blending of plastic granules with graphene nanoparticles by an extruder always leads to agglomeration and poor dispersion owing to the high melt viscosity [3]. Mechanically noncovalent mixing also results in poor interaction between fillers and polymer matrix. To overcome this problem, the in situ polymerization technology has emerged and displayed more advantages in the large-scaled production of nanocomposites with better dispersity in the form of covalent coupling [4]. In recent years, the chemical functionalization of nanoparticles has been used as reactive nanofillers for the production of high-performance polymer composites [5]. For example, the graphene particles were usually chemically functionalized via controlled edge oxidation to provide the reactive carboxyl groups on nanofillers [6].

Poly(ethylene terephthalate) (PET) is one of the most widely used thermoplastic polymers. It was used in a variety of types from water bottles to product packaging [7]. The melt-spun PET fiber was regarded as the most common polyester fiber used in textile industry due to its low production cost and excellent properties [8]. In recent years, PET filled with layered nanomaterials has been attracting intensive attention because of greatly improved performance, such as mechanical properties and impermeability of gas [9]. Wang et al. prepared a novel graphite nanoplatelet/PET nanocomposite for construction of melt-spun fibers. Compared to native PET material, the hybrid nanocomposites were found to decrease volume resistivity, which reinforcing its potential to be used in antistatic textile [10]. Salavagione and co-workers developed a mechanically stable and conductive graphene/PET textile by the dry-jet wet-spinning method from nanocomposite solutions in trifluoracetic acid [11]. The inhibition of microbial strains was another interesting ability of graphene-based polymer nanocomposites [12]. Currently, graphene/polymer nanocomposite films and coatings designed to inhibit bacterial growth are being explored and investigated for the fabrication of antibacterial food packaging and protective clothing [13]. However, a few works focus on the applications of fabric materials with antibacterial character.

Furthermore, safe and intelligent packaging materials for food storage and transportation have attracted growing interest in recent years [14]. Fruits have a limited postharvest lifespan due to their high nutritional and moisture content, making them susceptible to decay and spoilage. Over one-third of fruits spoil annually, resulting in economic losses amounting to several billions [15]. Functional packaging materials play a crucial role in maintaining the freshness of harvested fruits and extending fruit aging, which can generate significant economic benefits [16]. At present, addition of antimicrobial agents into packaging materials is usually used for inhibiting bacterial growth and preserving food quality [17,18,19,20]. The enhanced thermal/electrical conductivity of 2D nanofiller-modified composites also improves thermal management during storage and enables potential smart packaging applications [21]. Incorporation of graphene nanosheets not only provides antibacterial properties but also enhances oxygen and moisture barrier performance, thereby maximizing food preservation. In addition, MXene-based nanocomposites show exceptional antibacterial activity and barrier properties, setting new benchmarks for preservation materials [22]. However, graphene and graphene-based nanocomposites remain advantageous for scalable production. Unlike polylactic acid/carbon nanotubes/chitosan composite fibers by electrospinning process [23], melt-spun graphene/PET fibers offer intrinsic, non-leaching antibacterial activity by in situ polymerization technology.

An ideal fruit packaging material should be cost-effective, non-toxic, environmentally friendly, and possess both antibacterial and breathable properties [24]. Fruits naturally produce carbon dioxide during storage, and excessive sealing with poor breathability can accelerate decay. In fact, a proper oxygen gas exchange can suppress the growth of anaerobic bacteria. Conventional packaging films often suffer from inadequate breathability, while sprayed preservative coatings often leave residues due to incomplete cleaning [25]. Therefore, developing an antibacterial, breathable, lightweight and soft textile-based material holds great promise for improving fruit preservation, helping to maintain its quality and nutritional value. Unlike monolithic films, the woven structure of graphene/PET fabric naturally enables gas exchange and humidity control through interfiber gaps, preventing anaerobic conditions while maintaining antibacterial efficacy. In this work, we utilized in situ polymerization to covalently incorporate carboxylated graphene into PET, achieving superior dispersion and interfacial bonding compared to traditional melt-blending methods. Thereafter, the graphene/PET fibers were spun with the polymerized nanocomposites through a double-screw melt spinning machine. The resulting nanocomposite fibers exhibit excellent thermal stability, reduced crystallization defects, and significant antibacterial activity against *Escherichia coli* and *Proteus vulgaris*. The graphene/PET fabric extended the lifetime of strawberries, demonstrating its potential as an eco-friendly, breathable, and non-toxic packaging material for perishable foods.

## 2. Results

### 2.1. Preparation of Graphene/PET Nanocomposite Fibers

**Figure 1:**
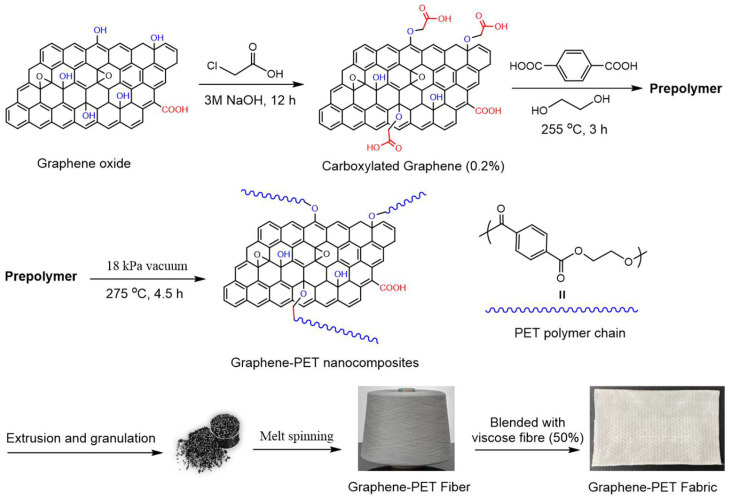
Synthesis of graphene/PET nanocomposites by in situ polymerization.

Currently, graphene-based nanocomposite materials in use are mostly prepared by physical mixing, in which the polymers and graphene are directly mixed through mechanical processing. Mechanical mixing is easy to operate. However, this method always results poor dispersity of nanofiller. In situ polymerization is an effective alternative technique for the formulation of highly dispersed graphene in a polymer matrix. This process involves the polymerization of monomers in the presence of the reactive layered materials. The in situ co-polymerization technology was therefore applied in our work, affording the covalently coupled graphene/PET nanocomposites. In detail, the carboxyl-functionalized graphene (0.2 wt%) as a crosslinking agent was reacted with EG and PTA to generate the in situ polymerization products. After extrusion, granulation, and melt-spinning processes, the final nanocomposite fabric was fabricated (Figure 1). According to the chemical structure of graphene oxide, more carboxyl groups were covalently modified on its surface to provide the reactive sites for the subsequent polycondensation. Furthermore, the functionalized graphene sheets were homogeneously dispersed in PET matrix through the in situ generation of grafted PET arms on graphene surfaces. Finally, the nanocomposite fibers and the flowing fabric were prepared by melt-spinning process with 0.2 wt% graphene loading. Herein, it should be noted that higher loading of graphene will negatively impact melt spinning, including spinneret blockage. During the melt spinning process, the melt pump pressure was consistently maintained below 20 MPa. This excellent spinnability, in turn, demonstrates the good dispersion of graphene particles in the PET matrix.

### 2.2. Structure and Morphology of the Nanocomposite Fibers

**Figure 2:**
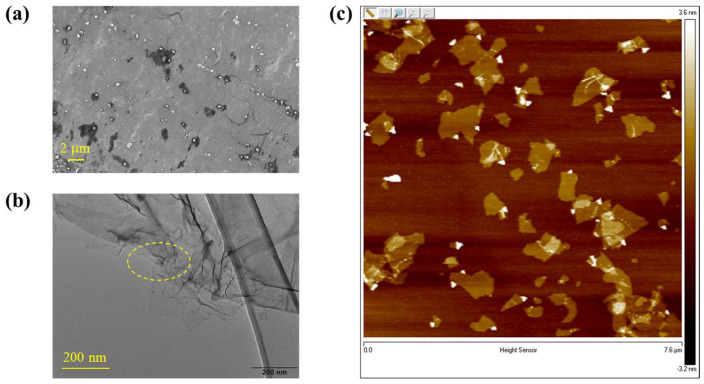
The micrographs of the carboxylated graphene samples: (**a**) SEM image, (**b**) TEM image, (**c**) AFM height image.

Typical micrographs of the carboxylated graphene samples are shown in Figure 2, which appears to have some very thin platelets present in the SEM images (Figure 2a), showing the typical 2D structure of graphene samples. As shown in Figure 2b, the TEM image gave a more distinct observation of the nanosheet morphology of the carboxylated graphene used in our experiment. AFM images of graphene were recorded and presented in Figure 2c to further observe its morphology and the thickness. It was shown that the vertical distances of the edge of dispersant-free suspension were 1.3 nm, which is characteristic of a single-layer graphene nanosheet.

**Figure 3:**
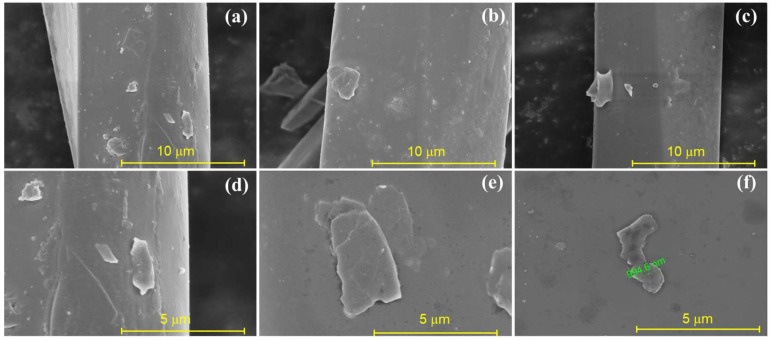
FE-SEM images of (**a**–**c**) surface and (**d**–**f**) magnified morphologies of graphene/PET nanocomposite fibers.

To characterize the morphologies of melt-spun graphene/PET fibers, FE-SEM images of the surfaces of PET fibers were examined. As shown in Figure 3, the relatively rough surface was noted on the surface of nanocomposites (Figure 3a–c). The magnified imagines were further recorded to reveal the nanosheet structure of graphene on the fiber surface (Figure 3d–f). The antibacterial performance can be attributed to this unique nanocomposite structure with a contact antibacterial mechanism [26].

**Figure 4:**
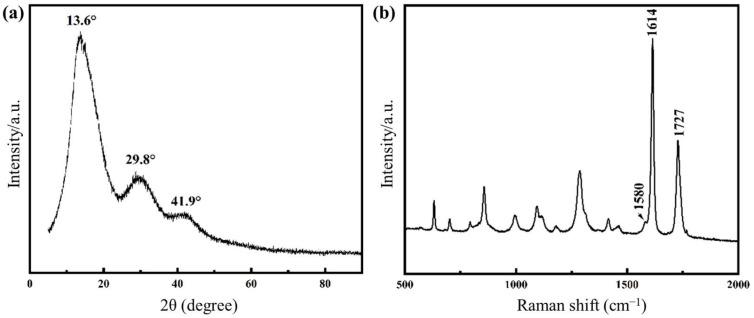
XRD and Raman survey of graphene/PET nanocomposite: (**a**) XRD pattern, (**b**) Raman spectrum.

The presence of graphene in PET composites was further revealed by XRD patterns (Figure 4a) and Raman spectroscopy (Figure 4b). The fiber sample exhibited a broad characteristic diffraction peak at 2θ = 13.6°, corresponding to the (001) crystal plane of GO. The peaks at 29.8° and 41.9° were attributed to the (002) plane of GO [27]. These results confirm the presence of graphene in the PET fiber sample. In addition, the typical structure of the graphene can be reflected in the Raman spectra (Figure S1), in which the G peak as the feature at 1584 cm^−1^ and a D band at 1361 cm^−1^ are present [28]. Herein, the G peak (Graphitic peak) and D peak (Disordered peak) in Raman spectroscopy are key characteristic peaks for analyzing the structure of graphene. They reflect the degree of carbon atom ordering and defects in the material. Specifically, the G peak indicates the graphitic ordered structure, while the D peak primarily reflects structural disorder, such as in graphene oxide where the D peak is prominent. In contrast, monolayer graphene exhibits a strong G peak. As shown in the Figure 4b, the fiber sample exhibits multiple vibration peaks, suggesting the possible presence of various substances. Among them, the peak near 1580 cm^−1^ corresponds to the G-band of carbon materials, indicating the existence of graphene sheets. The peak around 1614 cm^−1^ is characteristic of peaks of aromatic structures, likely attributable to PET chains. Additionally, the peak at 1727 cm^−1^ is assigned to the C=O (carbonyl) stretching vibration, which may arise from esters or carboxylic acids present in the sample. These results also suggest that the PET fiber sample contains trace amounts of graphene.

### 2.3. Thermal Analysis of the Nanocomposite Fibers

**Figure 5:**
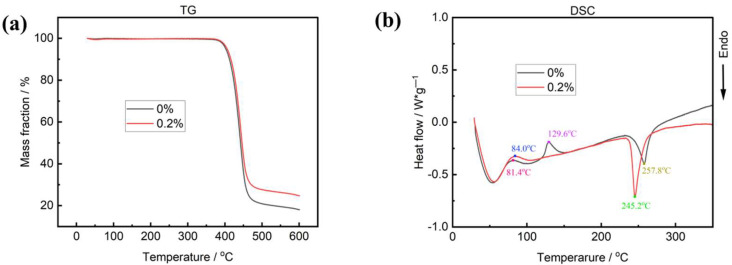
Thermal analysis for graphene/PET materials and PET materials: (**a**) thermal degradation curves; (**b**) DSC thermograms.

It is noteworthy that the thermal stability of nanocomposite was similar to that of pure PET. Thermal analysis curves of PET and graphene/PET samples are presented in Figure 5. The TG curve (Figure 5a) of PET exhibited significant weight loss between 400 and 450 °C, which was assigned to thermal decomposition [29]. The smaller weight loss of graphene/PET was attributed to the hybrid graphene particles in polymer matrix. The DSC curve of two samples, presented in Figure 5b, shows an exothermic peak at ca. 130 °C assigned to PET crystallization and the endothermic peaks at ca. 245 °C and 257 °C assigned to the melting of the graphene/PET and PET polymers, respectively. Semi-crystalline PET can undergo a process during a thermal ramp known as cold crystallization, which results in an exothermic peak above the Tg but below the melt Tm [30]. The high degree of amorphous content of PET can be observed by the relatively large cold crystallization peak. This indicates the large volume of amorphous polymer which was available to align and crystalize during the thermal ramp. It is interesting to note that the peak of cold crystallization disappeared for the graphene-hybrid PET samples. Compared to unmodified PET, the molecular chain regularity of graphene-modified PET is disrupted due to its lower chain flexibility, which also cause its lower melting point. This accelerates the mobility and motion speed of the molecular chains, leading to faster crystallization rates. Under the same cooling crystallization rate, the molecular chains of graphene-modified PET can more completely embed into the crystal lattice, and the polymer chains have sufficient time to adjust their conformations and fully align into the lattice, resulting in fewer crystallization defects and making the melting process less prone to cold crystallization [31]. Therefore, compared to unmodified PET, the cold crystallization peak disappears in graphene-modified PET. While graphene oxide (GO) has been widely studied in PET composites, its dispersion remains challenging via conventional melt blending. Our in situ polymerization technique ensures uniform GO distribution. Notably, the suppressed cold crystallization contrasts with nucleation-dominated effects reported in physically blended systems, suggesting that grafted PET chains restrict molecular mobility.

### 2.4. Antibacterial Activity and Fruit Preservation Using the Nanocomposite Fabric

**Figure 6:**
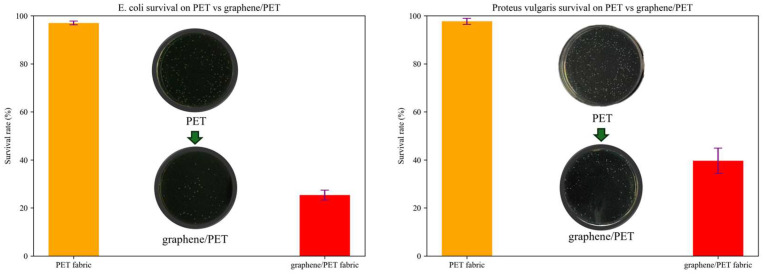
Antibacterial properties of graphene/PET materials and pure PET materials.

As mentioned previously, one of the primary purposes of adding graphene into the polymer matrix is to enhance the antibacterial activity of their generated nanocomposites [32]. To assess its antibacterial properties, two kinds of representative strain including *Escherichia coli* (EC) and *Proteus vulgaris* (PV) were used as the model bacteria. As shown in Figure 6, graphene/PET fiber exhibits an antibacterial ability toward EC and PV. To be more specific, nanocomposite fiber killed ~70% EC, and ~60% PV. Uniform dispersion of graphene in the PET fiber brought the attractive antibacterial properties of textile materials, which thereby favor packaging material for fruits. In comparison, pure PET fiber fabric exhibits no antibacterial activity.

**Figure 7:**
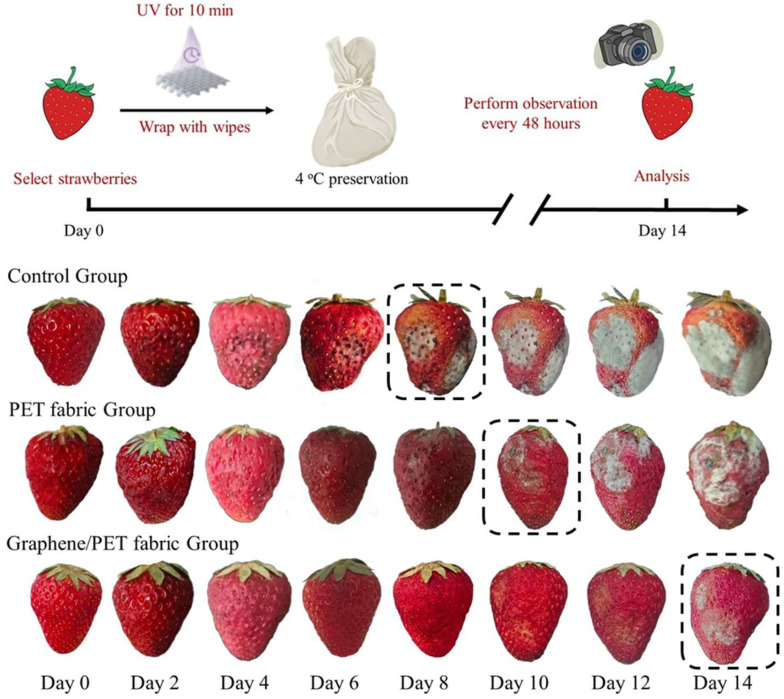
Photos of different fruits uncovered or covered with PET fabric, or graphene/PET fabric, respectively, for 14 days at 4 °C conditions.

Next, the graphene/PET fabrics were used for strawberry preservation. Fresh strawberry samples were divided into three groups and stored under refrigerated conditions. Among these, the control group consisted of unwrapped samples, the second group was wrapped with plain PET fabric, and the third group was wrapped with graphene/PET fabric. Observations and photographs were taken at 24 h intervals. The appearance and quality changes of strawberry during storage for 14 days at 4 °C were studied. As shown in Figure 7, the appearances of the strawberries packaged with graphene/PET fabric were much better than others. Specifically, the strawberry samples were individually sealed with different types of fabrics and stored under refrigeration. The antimicrobial activities of GO and its composite have been well reported through the production of highly reactive oxygen species (ROS) under light or in aqueous environments [33]. Prior to packaging, the fabrics were exposed to UV irradiation for 10 min to enhance their antibacterial properties, as graphene nanocomposites exhibits significantly improved antimicrobial activity under UV-induced oxidative stress [34]. In the control group (unwrapped strawberries), visible mildew and spoilage appeared at day 8. The group wrapped with unmodified PET fabric began showing mildew at day 10, whereas the graphene-modified polyester fabric delayed spoilage until day 14. Obviously, the antibacterial effect of the fabric can better resist the infection of foreign pathogens. These results demonstrate that the in situ polymerized graphene/PET fabric effectively inhibits microbial growth and significantly extends the shelf life of strawberries.

## 3. Materials and Methods

### 3.1. Chemicals

Terephthalate acid (PTA) was provided by Hengyi Petrochemical Co., Ltd. (Hangzhou, China). Ethylene glycol (EG) was supplied by Liaoyang Petrochemical Co., Ltd. (Liaoyang, China). Graphene oxides were offered by Jinan Shengquan Group Holding Co., Ltd. (Jinan, China). The recycled PET granules were supplied by Jiangsu Shenghong Petrochemical Group (Suzhou, China). Other commercially available compounds were used without further purification.

### 3.2. Synthesis of Graphene/PET Nanocomposites

The carboxylation of graphene oxide (GO) was carried out firstly to increase the amount of reactive sites on the surface of graphene for the next copolymerization [35]. Briefly, the GO (2.0 g) was dispersed in 3M sodium hydroxide solution (10 mL) followed by the addition of chloroacetic acid (1.8 g). The resulting suspension was bath sonicated for 2 h. The mixture was then stirred at room temperature overnight and filtered. The GO was collected and dispersed in water (10 mL), sonicated in a water bath and filtered again. This sequence was repeated 3 times with water and 3 times with methanol. The resulting solid was dispersed in water and the suspension was dialyzed in water for 3 days. After lyophilization, carboxylated graphene (cGO) was obtained. The functionalized cGO was pre-dispersed in the EG solution by constant ultra-sonication. The polycondensation was carried out in a 5L reactor with the feed molar ratio of EG:PTA:cGO (1.6:1.0:0.002) in the presence of titanium catalyst (0.06 wt%). The resulting mixture was heated to 255 °C to allow the esterification reaction to proceed for 3 h. Next, vacuum was applied and adjusted to gradually decrease the pressure to 18 kPa while the reaction temperature was increased to 275 °C for 4.5 h until the intrinsic viscosity reached 1.08 dL/g. Finally, the graphene/PET nanocomposites were obtained through extrusion and granulation for manufacturing polyester yarns (Figure 1). After, the extruder barrel was heated to the temperature of 270 °C. The graphene/PET was maintained in the barrel at 270 °C with very low screw speed for 15 min to achieve a complete melting. The screw speed of the extruder was adjusted to 10 rpm in order to obtain a homogenous spread of melted PET in the extruder barrel. Then, the speed of the screw was increased to 50 rpm to extrude the entire PET from the extruder.

### 3.3. Characterization Methods

Intrinsic viscosity was tested by a Ubbelohde viscometer at 25 °C with a mixture of phenol and 1,1,2,2-tetrachloroethane (1:1, *w*/*w*) as solvent. AFM was recorded on a Bruker atomic force microscopy (AFM) in tapping mode. Differential scanning calorimetry (DSC) was performed on TA Q2000 (New Castle, DE, USA) using nitrogen gas blowing, with a temperature range of 30 to 350 °C and a heating rate of 10 K/min. TGA was performed on Rigaku TG/DTA 8122 (Akishima, Japan). Samples were heated from 30 °C to 600 °C at a heating rate of 20 °C/min under a nitrogen atmosphere. The morphologies of graphene/PET nanocomposite fibers were observed using a Nova NanoSEM 450 scanning electron microscope (FEI, Hillsboro, OR, USA). The samples were coated with gold using a sputter coater. TEM was recorded on the ThermoFisher Scientific (Talos F200S, Waltham, MA, USA). Raman spectroscopy was recorded using LabRAM Odyssey (Horiba, Kyoto, Japan). XRD was characterized using Smartlab SE (Rigaku) which was operated with Cu Ka radiation (k = 1.5405 Å) at 40 kV and 30 mA. Fourier transform infrared spectrometer (FT-IR) was recorded on Bruker ALPHA II (Billerica, MA, USA).

### 3.4. In Vitro Antibacterial Assay

The *Escherichia coli* (EC) and *Proteus vulgaris* (PV) were obtained from BeNa Culture Collection (BNCC, Kunshan, China). The bacteria were cultured in a Luria–Bertani (LB) medium for 24 h at 37 °C, 150 r/min. For antimicrobial test, the bacterial suspension after gradient dilution was co-incubated for 0.5 h at 37 °C with 5.0 mg of fabric sample (ultraviolet irradiation for 10 min), 20% sodium hypochlorite solution as the positive control, and normal saline as the negative control. Then, the bacterial suspension was cultured for 24 h, and the CFU count was performed.

## 4. Conclusions

In this work, the graphene/PET nanocomposite fibers were successfully produced by in situ polymerization of EG and PTA in the presence of carboxylated graphene (cGO). The efficient polymer-chain grafting achieves good dispersion of nanoparticles in PET matrix. The homogenous PET matrix nanocomposites containing 0.2 wt% graphene showed a good spinnability in the melt spinning process. Covalent grafting of PET chains onto cGO nanosheets ensures uniform dispersion in the PET matrix, overcoming aggregation issues of melt blending. Furthermore, we prepared graphene/PET fibers by melt-spinning process to produce the antibacterial PET fibers. The obtained nanocomposite textiles exhibit >60% bacterial reduction via contact-killing and ROS generation. It was noted that graphene/PET fabrics can significantly extend the shelf life of strawberries to 14 days at 4 °C. In comparison, unwrapped strawberries last only 8 days, while those wrapped in pure PET only remain fresh for 10 days. This work establishes a strategy for designing high-performance nanocomposites where covalent integration of functionalized nanofillers optimizes structural, thermal, and antimicrobial properties. Further applications of the graphene-hybrid fibers and preparation to other graphene-modified polymer composites by in situ polymerization are in progress.

## Data Availability

All the relevant data that support the findings of this study are available from the corresponding author on reasonable request.

## Supplementary Materials

The following supporting information can be downloaded at: https://www.mdpi.com/article/10.3390/molecules30153109/s1. Figure S1: Raman spectra of the carboxylated graphene; Table S1: Analysis of carboxyl group content in graphene samples; Figure S2: DTG spectra of the graphene/PET nanocomposite fiber; Table S2: Physical properties of the graphene/PET nanocomposite fiber; Figure S3: Optical microscopy images of the nanocomposite fibers; Figure S4: TEM images of the graphene structures on nanocomposite fibers; Figure S5: Comparative infrared spectra of pure PET (0 %) and graphene/PET nanocomposite (0.2%).
