# Supplementary material for: Fabrication of Antibacterial Poly(ethylene terephthalate)/Graphene Nanocomposite Fibers by In Situ Polymerization for Fruit Preservation
Source: Molecules. 2025 Jul 24;30(15):3109. doi: 10.3390/molecules30153109 (PMC12348518; doi:10.3390/molecules30153109)
Supplement: Supplementary file 1 [file molecules-30-03109-s001.zip › molecules-3731900-supplementary.pdf]

Fabrication of antibacterial Poly(ethylene terephthalate)/Graphene nanocomposite fibers by in-situ polymerization for fruit preservation

Jiarui Wu [1], Qinhan Chen [1], Aobin Han [1], Min Liu [1], Wenhuan Zhong [1], Xiaojue Shao [2], Yan Jiang [2], Jing Lin [3], Zhenyang Luo [1], Jie Yang [1,*], and Gefei Li [1,*]

[1] College of Science, Nanjing Forestry University, Long Pan Road No.159, Nanjing 210037, P. R. China; gefei@njfu.edu.cn

[2] Highbery New Nano Materials Technology Co.,Ltd., Changzhou 213000, P. R. China; jy@highbery.cn

[3] Nanjing Institute of Metrological Supervision and Testing, Ma Qun Avenue No.10, Nanjing 210049, P. R. China; linjing009@126.com

* Correspondence: jieyang@njfu.edu.cn (JY); gefei@njfu.edu.cn (GL)

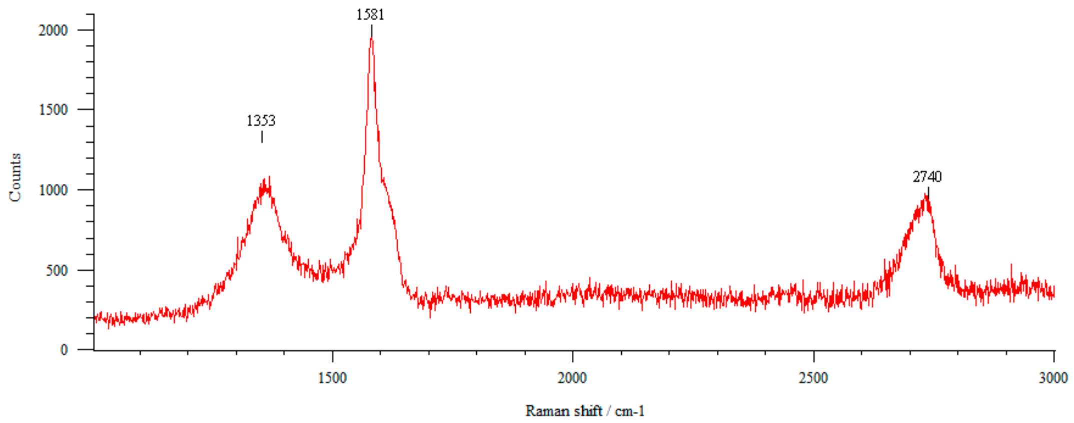

Peak no.	Centre	Height	Width	Area	Absolute intensity	Low edge	High edge
1	1353.3	961.496	53.2798	242609	1301.91	1184.8	1458.74
2	1580.6	1563.12	31.3538	236432	1965.95	1484.08	1686.58
7	2739.53	-	-	-	951.948	-	-

Figure S1. Raman spectra of the carboxylated graphene

Table S1. Analysis of carboxyl group content in graphene samples

**Sample**	**Carboxyl group content (mmol / g)**
Graphene oxide	**2.1**
Carboxylated graphene	**4.94**

Figure S2. DTG spectra of the graphene/PET nanocomposite fiber

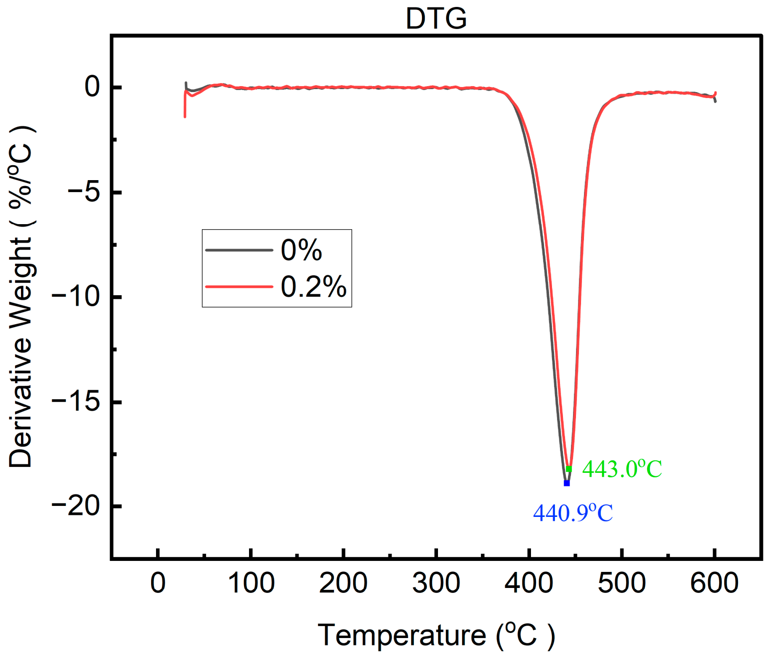

Table S2. Physical properties of the graphene/PET nanocomposite fiber

Mn	Viscosity	Surface resistivity	Tensile stress	Ultraviolet Protection Factor (UPF)
24500	1.08 dL/g	8.0×10^{11} Ω	6.7 cN/dtex	>50

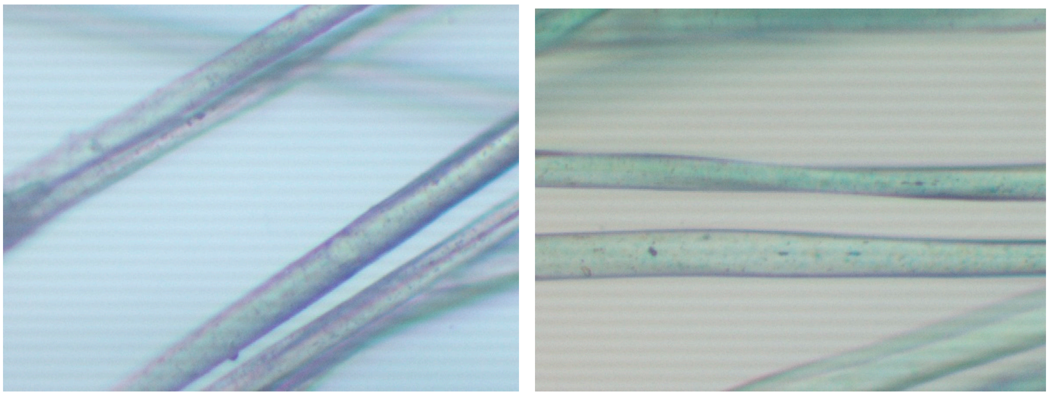

Figure S3. Optical microscopy images of the nanocomposites fibers

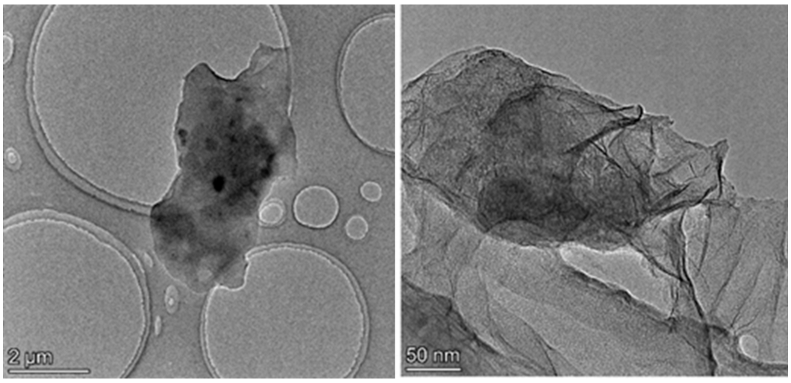

Figure S4. TEM images of the graphene structures on nanocomposite fibers

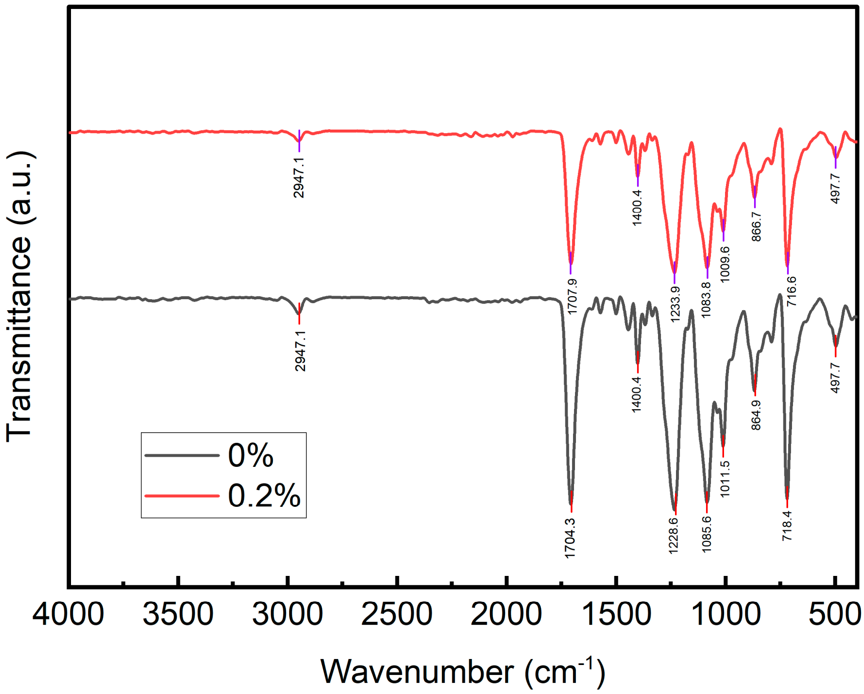

Figure S5. Comparative infrared spectra of pure PET (0 %) and graphene/PET nanocomposite (0.2%): because the carboxyl groups and the *in situ* formed ester bonds on the graphene are completely identical to those in PET, the IR spectroscopy does not show significant differences.
